# Supplementary material for: Metabolomic response of Euglena gracilis and its bleached mutant strain to light
Source: PLoS One. 2019 Nov 7;14(11):e0224926. doi: 10.1371/journal.pone.0224926 (PMC6837420; doi:10.1371/journal.pone.0224926)
Supplement: Attachment — Submitted filename: Responses to editor and reviewers.docx [file pone.0224926.s002.docx]

Reviewer #1

*1.Questions: The last paragraph of the introduction, no result should be presented or discussed in the introduction.*

Response: Thanks for your constructive advices. We have moved the sentence “the relatively significant differences in either response time or metabolite components between WT and OflB2 under light stimulation” from introduction into discussion (lines 189-190).

*2.Questions: Line 103, what does it mean "The solvent was delayed by 10 min"?*

Response: Sorry for the confusion caused by the unclear description. We changed the "The solvent was delayed by 10 min" to "Metabolites retained in capillary column for at least 10 minutes"(lines 95-96).

*3.Questions: Line 191, why the low growth of growth of the OflB2 mutant is unexpected? this needs better explanation.*

Response: Since WT Euglena could grow heterotrophically and autotrophically under different culture conditions, and in the dark WT Euglena cannot photosynthesize and the chloroplasts inside cells may be a burden for cells. Without chloroplasts in the dark, the mutant cells were expected to grow better than WT since they have no material and energy waste for chloroplasts. We have added this paragraph into the revised manuscript (Line 159-162).

*4.Questions: It is hard for readers to understand when conclusion is made from unpublished data. Suggest to delete or modify the 2nd paragraph of the summary.*

Response: Thanks for useful suggestions. We have deleted the conclusion based on unpublished data.

Reviewer #2

*1.Questions: Figure 1 cannot be found.*

Response: Sorry for low mistake by the first time submissioner. We have added figures in the revised version and this time we promise every figure are included.

*2.Questions: Morphological changes of chlorophyll and other physiological indicators should be observed*

Response: in the new version we added and analyzed the differences of pigments between wild type and the bleached mutant in lines 121-125

*3.Questions: Add GC-MS Analysis of Raw Data Graph.*

Response: we have added a supplementary material containing the raw analysis data of GC-MS in the newly revised version.

*4.Questions: It is suggested that genomics, transcriptome and proteomics data be published together to better reveal the mechanism of light effects on them.*

Response: thanks for constructive advice. We agree completely with you. We have the RNA-seq and proteomics data but still wait for the ongoing Euglena genome assemble, to make a better annotation and analysis.

*5.Questions: There are some grammatical errors in the manuscript, please modify the language further.*

Response: we have corrected the grammatical errors as possible as we can. Please check for the details in the revised manuscript.

*Academic Editor：*

*We will only publish funding information present in the Funding Statement section of the online submission form.*

*Please amend your list of authors on the manuscript to ensure that each author is linked to an affiliation.*

Response: we have deleted the funding information from the manuscript text, as request. We amended the list of authors and ensure that each author has only one major affiliation.
